# Adaptive Behavior and Bullying Experiences in Spanish-Speaking Children and Adolescents with Autism Spectrum Disorder Level 1

**DOI:** 10.3390/children12121707

**Published:** 2025-12-17

**Authors:** Alberto Sánchez-Pedroche, Daniel Adrover-Roig, Mario Valera-Pozo, María Fernanda Lara Díaz, Eva Aguilar-Mediavilla

**Affiliations:** 1 Department of Applied Pedagogy and Educational Psychology, Institute of Research and Innovation in Education (IRIE), University of Balearic Islands, 07122 Palma, Spain; daniel.adrover@uib.es (D.A.-R.); m.valera@uib.es (M.V.-P.); eva.aguilar@uib.es (E.A.-M.); 2 Antic Edifici de la Comandància Carrer del Calvari, 1, Universitat de les Illes Balears, Seu Eivissa, 07800 Eivissa, Spain; 3 Human Communication Department, Faculty of Medicine, Universidad Nacional de Colombia, Bogotá 111321, Colombia; depcomhd_bog@unal.edu.co

**Keywords:** bullying, victimization, aggression, Autism Spectrum Disorder (ASD-L1), adaptive behaviors, maladaptive behaviors

## Abstract

**Highlights:**

**What are the main findings?**

• Children with ASD-L1 reported higher levels of clinical and school maladjustment and aggression and lower personal adjustment compared to controls, but no significant differences in victimization were observed.
• While lower personal adjustment predicted bullying victimization, larger clinical maladjustment predicted aggression in the ASD-L1 group.

**What is the implication of the main finding?**

• Clinical maladjustment was identified as a risk factor for bullying involvement among children with ASD-L1, whereas school maladjustment predicted aggression among typically developing peers
• Interventions promoting emotional regulation, interpersonal abilities, and self-esteem may be particularly relevant for reducing aggression and victimization in this vulnerable population.

**Abstract:**

Background/Objectives: This study aims to compare adaptive and maladaptive behaviors of children with Autism Spectrum Disorder Level 1 (ASD-L1) and their experiences of bullying in comparison to a matched control group. Additionally, we explored which of such behaviors predicted both victimization and aggression in both samples. Methods: The sample consisted of 96 children and adolescents, 48 with ASD-L1 (31 Colombians and 17 Spanish) and 48 controls (31 Colombians and 17 Spanish), matched by age, gender, and socioeconomic status. Adaptive and maladaptive behaviors, as well as bullying experiences, were assessed. Results: Children with ASD-L1 reported higher levels of clinical and school maladjustment and lower levels of personal adjustment compared to the control group. Although no significant differences were found in bullying victimization, the ASD-L1 group showed higher aggression scores. In this group, lower personal adjustment predicted victimization, whereas higher clinical maladjustment predicted aggression. In contrast, in the control group, aggression was predicted by school maladjustment. Conclusions: Aggressive behavior in children with ASD-L1 was linked to higher levels of clinical maladjustment, while better personal adjustment served as a protective factor against bullying victimization. These findings emphasize distinct socio-emotional mechanisms underlying bullying involvement in autistic and typically developing youth.

## 1. Introduction

Autism Spectrum Disorder (ASD) is a neurobiological condition characterized by persistent deficits in social communication and interaction, as well as restrictive and repetitive patterns of behavior, interests and/or activities [1,2]. All these deficits must be present since childhood and cause significant functional impairment. ASD is characterized by a range of symptoms and levels of severity, as defined by the DSM-5 TR [1].

For the present study, we focus on participants with ASD Level 1 (ASD-L1), who need some support but typically do not experience significant difficulties with language or self-care skills [3]. This diagnostic category broadly corresponds to what previous literature has referred to as Asperger’s syndrome or high-functioning autism (HFA), prior to the DSM-5 diagnostic shift [4]. Whilst a substantial corpus of literature has examined Autism Spectrum Disorder (ASD) as a broad and heterogeneous category, there is an increasing need to study the specific socio-emotional and communicative profiles of ASD Level 1 (ASD-L1). Consequently, the present study contributes to this emerging line of research by focusing specifically on ASD-L1 rather than ASD in general.

In this vein, individuals on the autism spectrum (particularly those with Level 1) face a range of socio-emotional challenges that can vary widely between individuals. Difficulties in non-verbal communication and in understanding social cues may lead to misunderstandings in social interactions [5]. They also tend to experience challenges in identifying, understanding, and expressing their own emotions, as well as in recognizing and responding to the emotions of others [5,6]. Such difficulties often affect emotional self-regulation and stress management, with implications for overall well-being [6]. Furthermore, while individuals with ASD may wish to establish and maintain social relationships, they often struggle with social rules and may experience social isolation as a result [7,8].

Similarly, individuals diagnosed with ASD-L1 are expected to manifest restrictive and repetitive behavioral patterns, interests, or activities, as these behaviors are identified as core diagnostic features across the entire spectrum of ASD [1]. Although these behaviors may not be as prominent as in more severe cases of ASD, they may still limit participation in social activities and the ability to adapt to new situations. Difficulties in emotional regulation, social understanding, and interpreting others’ emotions are core socioemotional features observed across the autism spectrum, including individuals with Level 1 profiles. Such factors have been shown to influence developmental trajectories and social adjustment over time [9]. Individuals with ASD-L1 often experience challenges in understanding and engaging in meaningful social interactions, which can lead to social anxiety and feelings of isolation or loneliness [10].

In addition, difficulties in verbal and non-verbal communication may hinder the expression and understanding of emotions, which may contribute to emotional regulation problems [7]. These challenges can create stress and lead to a wide range of emotional and behavioral responses, including aggression, social withdrawal, and emotional dysregulation, which may interfere with day-to-day functioning [11].

This type of response is considered maladaptive and may occur in various areas of daily life, including family, school, and social settings. Studying these variables allows for a more precise understanding of the emotional and social functioning of children and adolescents diagnosed with ASD-L1. In this sense, it is crucial to study the social-emotional factors in individuals with ASD-L1 to improve their treatment and support and mitigate the challenges associated with their difficulties and improve their quality of life [9].

### 1.1. Maladaptive and Adaptive Socio-Emotional Functioning in ASD-L1

In order to comprehend the vulnerability of students with ASD-L1, it is crucial to differentiate between maladaptive and adaptive functioning. Within the parameters of this study, maladaptive functioning is defined as behaviors or symptoms that impede social integration and personal well-being, operationalized through two distinct composite dimensions [11,12]. First, Clinical Maladjustment captures internalizing symptoms and atypical behaviors, encompassing scales such as anxiety, atypicality, and locus of control. This dimension is particularly relevant for the ASD-L1 profile, as previous research suggests that emotional dysregulation and internal distress are core features that may predispose these students to reactive aggression [13,14]. Second, School Maladjustment reflects dissatisfaction with the educational context, including negative attitudes towards school and teachers.

Conversely, the concept of adaptive functioning, conceptualized here as Personal Adjustment, encompasses the student’s psychological resources and resilience. This dimension integrates key strengths such as self-esteem, self-reliance, and the quality of interpersonal relations with parents and peers [11,12]. Assessing these adaptive skills is crucial, as they have been identified as potential protective factors against bullying victimization in adolescents with autism [15,16]. By examining both maladaptive and adaptive domains, this study aims to provide a comprehensive picture of the socio-emotional mechanisms underlying bullying involvement in ASD-L1.

### 1.2. Relationship Between ASD and Bullying

Because of socio-emotional difficulties and the traits of ASD [17], individuals with ASD-L1 might be more likely to experience bullying. Bullying refers to aggressive and repetitive behaviors that occur in a context where there is an imbalance of power, either real or perceived, between the aggressor and the victim [18].

Bullying manifests in various forms, including physical, verbal, and relational aggression, both in-person and online [19]. Despite the implementation of anti-bullying protocols, it remains a pervasive issue in schools [20], with behaviors like teasing, exclusion, and threats [21]. Bullying affects not only victims but also bystanders and aggressors, causing emotional, psychological, and academic harm [22]. Addressing bullying requires a comprehensive approach involving awareness, prevention, and early intervention, supported by evidence-based policies and training for the school community [15,23].

A substantial body of research has repeatedly concluded that individuals diagnosed with ASD are considered to be a particularly susceptible demographic group regarding victimization by peers. While the current diagnostic framework (DSM-5-TR) maintains the severity levels introduced by the DSM-5 over a decade ago, recent evidence has begun to specifically address the ASD Level 1 profile. For instance, Ferrigno et al. [24] identified individuals with ASD Level 1 as the most frequent targets of bullying within the spectrum, representing 65% of all victimized autistic individuals in their sample. Similarly, Gkatsa and Antoniou [17] highlighted the heightened vulnerability of students with High-Functioning Autism in inclusive settings. These findings align with previous literature estimating victimization rates for these profiles between 50% and 75% [25,26]

Regarding the types of bullying, while physical bullying does occur, specific literature on these profiles points to a predominance of verbal and relational forms (e.g., social exclusion), which are often harder for teachers to detect but devastating for these students due to their greater social awareness and desire to fit in compared to profiles with greater severity [25,27].

Previous studies based on external informants have also found that students on the autism spectrum experience higher rates of bullying victimization compared to typically developing peers (hereafter, TD). For instance, Van Schalkwyk et al. [26] conducted a parent-reported study including children and adolescents with ASD, most of whom were diagnosed with Asperger’s syndrome or HFA and found that 51% reported recent bullying, with 31% of parents confirming victimization

Previous research has shown that students with ASD are more likely to experience bullying due to their social and communicative difficulties [28]. In particular, individuals with ASD-L1 often display distinctive behaviors and traits that increase their visibility and vulnerability among peers [24]. Social communication deficits, restrictive behaviors, and cognitive rigidity contribute to misunderstandings, social isolation, and difficulty in responding to bullying effectively [29]. Additionally, children with ASD frequently report feelings of loneliness and lower social competence, exacerbating their vulnerability to bullying [30].

The stress from bullying aggravates socio-emotional challenges in ASD, affecting emotional well-being [31]. Studies using the Behavioural Assessment System for Children (BASC) confirm that individuals with ASD, including those with HFA, experience significant emotional and social difficulties, such as heightened levels of hyperactivity, attention problems, and depression [13,32,33].

### 1.3. Relationship Between Socio-Emotional Functioning and Bullying Involvement

As previously stated, maladaptive and adaptive dimensions are significant factors in bullying dynamics. For instance, externalizing behaviors are often associated with higher levels of bullying perpetration. However, in the context of ASD, this aggression is frequently described as reactive rather than proactive, stemming from difficulties in emotion regulation and frustration management rather than intent to dominate [21].

Regarding victimization, internalizing symptoms such as anxiety and depression significantly increase its risk. Specifically, Chou et al. [34] found that adolescents with High-Functioning Autism Spectrum Disorder (HFASD) involved in bullying—particularly victims and perpetrator-victims—exhibit significantly higher levels of depression, anxiety, and suicidality compared to those not involved [34]. Furthermore, studies focusing on this specific profile have identified “social vulnerability” (defined by traits such as credulity and gullibility) as a stronger predictor of victimization than autism severity itself, suggesting that the inability to detect social deception makes these students easy targets [35]. Conversely, protective traits such as high self-esteem and robust interpersonal relationships have been demonstrated to reduce vulnerability to bullying [16].

### 1.4. Self-Reports in ASD

Most research on ASD has relied on assessments by parents or teachers, but self-reports provide unique insights, as individuals with ASD may perceive and interpret their social and emotional experiences differently than adults around them [36,37]. Studies have highlighted those children with ASD often self-report higher levels of internalizing symptoms, such as anxiety or depression, compared to assessments made by external informants, who may underestimate these difficulties [38]. This discrepancy underscores the value of self-reports in capturing aspects of emotional and social experiences that might otherwise be overlooked, thereby providing a more comprehensive understanding of their well-being.

In recent research, self-reports have proven to be effective in identifying specific emotional states and adaptive skills within the ASD population. For example, Bakhtiari et al. [39] demonstrated the reliability of self-report measures using the BASC in individuals with ASD, emphasizing the need to include their perspectives alongside those of parents and teachers. Furthermore, self-reports have been shown to be valuable tools for evaluating mental health aspects like anxiety, depression, and negative thought patterns, which may not be as readily observed by others [40]. Although self-reports pose certain challenges, they are crucial in capturing internal states that contribute to a holistic understanding of ASD. Therefore, this study emphasizes self-reported measures in a Spanish-speaking context, aiming to bring forward the voices of individuals with ASD-L1.

### 1.5. Objectives and Hypothesis

The present study is predicated on this framework, with the aim of analyzing the behavioral profiles in children and adolescents with ASD-L1 compared to their TD peers. Specifically, the objectives of this study are threefold: firstly, to compare self-reported adaptive and maladaptive behaviors between both groups; secondly, to examine group differences in self-reported bullying victimization and aggression; and thirdly, to explore the predictive role of the measured adaptive and maladaptive variables in bullying involvement.

In accordance with the extant literature, three specific hypotheses are hereby proposed. We first predicted that children and adolescents diagnosed with ASD-L1 would show higher levels of self-reported maladaptive behaviors (i.e., Clinical and School Maladjustment) as well as lower scores in variables measuring adaptive functioning (i.e., Personal Adjustment) in comparison to the TD group (H1). Moreover, we hypothesized that individuals diagnosed with ASD-L1 will report higher scores in variables measuring both victimization and aggression relative to TD peers (H2). Finally, given the specific vulnerability of this population, we anticipated that adaptive and maladaptive factors would be more closely linked to bullying involvement in this group, functioning as predictors or protective factors of both aggression and victimization (H3).

## 2. Materials and Methods

### 2.1. Participants

This study derives from an initial sample of 102 Spanish-speaking children with and without ASD-L1 with an average age of 10 years, hailing from Colombia (Bogotá) and Spain (Balearic Islands).

All children within the ASD-L1 group had received prior clinical diagnoses from their pediatric neurologist, adhering to established guidelines [4], and were assessed using standard clinical criteria. Thus, the clinical protocol for the diagnosis, treatment and comprehensive care pathway for children with ASD established by the Ministry of Health of the Colombian government [41] was followed for the Colombian sample. For the Spanish sample, the clinical protocol established by the Institute of Child and Adolescent Mental Health (IBSMIA) of the Autonomous Government of the Balearic Islands [42] was followed. Therefore, the ASD sample in our study consists of individuals diagnosed with level ASD-L1 (Requires Support), as defined by the DSM-5 TR [1]: Absence of intellectual disability (clinical documentation or estimated IQ ≥ 70); fluent verbal communication; and need for some support in social/pragmatic functioning as reported by professionals and/or caregivers. It is important to note that, in accordance with the DSM-5 criteria, the ASD-L1 group consisted of participants who, according to previous diagnostic classifications, would have been identified as having Asperger syndrome or HFA. It was determined that all participants satisfied the criteria for ASD-L1, thus substantiating the current study’s assertion that this diagnostic unification is a notable strength.

For every child in the ASD-L1 group, a control child was selected as similar as possible in terms of age, gender, and socio-demographic characteristics to the ASD participant.

To confirm the diagnosis reported by clinicians, our team administered the Autism Spectrum Screening Questionnaire (ASSQ; [43]), the Autism Spectrum Questionnaire (AQ; [44,45]) and the Raven test [46]. These instruments have been extensively utilized and have received substantial validation. As demonstrated in the original versions of each test, reported internal consistency indices (Cronbach’s alpha) are satisfactory: α = 0.89 for the ASSQ [43], α = 0.85 for the AQ-Child [44], and split-half reliability above 0.90 for the Raven’s Standard Progressive Matrices [46]

Three participants were excluded from the ASD-L1 group because their scores in the ASSQ and AQ tests did not meet the ASD criteria. Similarly, three participants in the control group were discarded from the sample, as two of them showed ASD behaviors according to the ASSQ and AQ tests, and the other had a very low IQ, which was not compatible with typical development.

The final sample was composed of 48 students with ASD-L1 (31 Colombians and 17 Spaniards) matched with 48 children acting as control group (31 Colombians and 17 Spaniards), see Table 1 for more information. Gender distribution did not differ between groups [*χ*^2^(1) = 0.154: *p* = 0.695], being only four female participants in each group, which is a common finding in ASD studies [47]. The age range of participants was 8–12 years (see Table 1) and according to the results of Raven’s test all the participants presented average cognition. The ASD and control group did not differ in the distribution of SES [*χ*^2^(4) = 3.99; *p* = 0.41] being this variable equivalent between groups (*t*(94) = −0.477, *p* = 0.634, *d* = 0.09). Therefore, none of the demographic variables showed differences between groups (see Table 1). Conversely, autism symptom questionnaires revealed significant differences between the ASD and control groups, with the average scores of the ASD-L1 group closely mirroring those of the validation sample.

All children had completed the previous two years of primary education and all participants in the ASD-L1 group demonstrated oral language abilities required to complete the various administered tests. Clinical professionals responsible for initial assessments excluded participants who were undergoing intensive psychiatric treatment or had received a comorbid diagnosis of a severe developmental disorder, in accordance with established clinical protocols.

### 2.2. Materials

*Behavioural Assessment System for Children* (BASC-second edition S2 and S3) [11,12].

The Behavioural Assessment System for Children (BASC) [11] provides a comprehensive assessment of behavior and adaptive functioning in children and adolescents. For the present study, we used the self-assessment forms of the Spanish version [12]: from 6 to 12 years (S2; 146 items) and from 13 to 18 years (S3; 185 items) with a dichotomous answer (yes/no).

In the present study, we focused on the global composite dimensions derived from the BASC, specifically those representing the clinical and adaptive domains. The clinical domain included School Maladjustment (negative attitudes toward school and teachers in the S2 form, plus sensation seeking in the S3 form) and Clinical Maladjustment (anxiety, atypicality, locus of control in the S2 form, plus somatization in the S3 form), while the adaptive domain was represented by Personal Adjustment (interpersonal relations, relationships with parents, self-esteem, and self-confidence).

After theoretical and empirical review of the BASC S2/S3 structure, two composites were excluded from the analyses. The Emotional Symptoms Index was removed due to its conceptual and statistical overlap with Clinical Maladjustment, as both share subscales such as anxiety and somatization. Likewise, the Triad (Stress–Anxiety–Depression) composite (derived from the Emotional Symptoms Index and not included in the original BASC manual) was excluded because it lacked conceptual independence and stability in the present data. Additionally, to ensure that scores on the BASC S2 and S3 versions could be compared, conceptually equivalent dimensions were identified and equated. This allowed them to be grouped and analyzed as a single set of constructs.

Consequently, three stable and non-overlapping composites (School Maladjustment, Clinical Maladjustment, and Personal Adjustment) were retained for analysis. Reliability coefficients from the Spanish validation [12] were satisfactory, ranging between α = 0.77 and α = 0.89 for the composite dimensions. In the current study, internal consistency coefficients were adequate for the composite dimensions Clinical Maladjustment (α = 0.93), School Maladjustment (α = 0.82), and Personal Adjustment (α = 0.79). These dimensions provided a coherent and reliable representation of adaptive and maladaptive functioning in the study samples.

*EBIP-Q (European Bullying Intervention Project Questionnaire)* [49,50].

We used the Spanish version of the European Bullying Intervention Project Questionnaire [50], which assesses bullying experiences. It is composed of seven items addressing self-perceived victimization and seven items evaluating self-perceived aggression. The ensemble of items comprises different forms of bullying behaviors. Examples of victimization items include verbal victimization (e.g., “Someone has insulted me”), physical victimization (e.g., “Someone has hit, kicked or pushed me”), instrumental victimization (e.g., “Someone has stolen or broken my things”) and relational victimization (e.g., “I have been excluded, isolated or ignored by other people”). Examples of aggression items include behaviors such as insulting others (“I have insulted and said offensive words to someone”), physical aggression (“I have hit, kicked or pushed someone”), threats (“I have threatened someone”), or relational aggression (“I have excluded or ignored someone” or “I have spread rumors about someone”). All items are answered using a five-level Likert scale with scores ranging from 0 to 4, which measures the frequency of the behaviors (from “never” to “more than once a week,” referring to the last 2 months). Reliability coefficients from the original validation were satisfactory (ω between 0.72 and 0.82) [51]. In the current study, reliability analysis yielded good internal consistency for both the Victimization (α = 0.83) and the Aggression (α = 0.79) subscales.

### 2.3. Procedure

The present study received approval from the Ethics Committee of both the University of the Balearic Islands and the National University of Colombia (Act No. 013-160-19). The project adheres to the ethical guidelines established by Resolution 008430 (4 October 1993) of the Colombian Ministry of Health and the Declaration of Helsinki principles for the protection of human participants. In addition, all parents of participants provided informed written consent.

All participants were recruited via collaborating schools and clinical institutions in Spain and Colombia that provided access to families of children with and without ASD-L1. Written informed consent was obtained in accordance with ethical guidelines, after providing all participants’ parents or legal guardians with the necessary information.

The assessments were carried out in two 45 min sessions, administered either at the university or the school in a similar way for both samples (Spain and Colombia). All instruments were administered individually by trained team members, ensuring adequate conditions for testing. In addition, no supplementary observers or caregivers were present during the sessions, and no audio or video recordings were made. All the data were collected directly from the participants’ responses, which were recorded on paper forms or digital platforms, and then subsequently anonymized for the purposes of analysis. During the administration of the self-report measures, verbal clarifications were provided to participants in the ASD-L1 group on a limited basis, solely in instances where uncertainty was observed. The purpose of these clarifications was to ensure comprehension of the content of the items, particularly regarding abstract emotional or social concepts. It should be noted that the items were not rewarded or altered as a result.

All responses were corrected and coded according to the established indications.

### 2.4. Data Analysis

The data were analyzed with JASP (version 0.17) [52] and SPSS (version 22). Independent samples tests were employed to compare the principal variables of the present study between groups. Specifically, Levene’s test confirmed that the assumption of homogeneity of variance was met for all analyzed variables (all *p*s > 0.05). Regarding normality, Shapiro–Wilk tests showed that Victimization scores followed a normal distribution (*p* > 0.05), whereas Aggression and the BASC scores showed a deviation from normality (*p* < 0.05). Therefore, we used *t*-tests for variables that followed a normal distribution, and Mann–Whitney *U* tests for variables that do not follow a normal distribution. Furthermore, Chi-square tests were employed to compare categorical variables between groups.

To examine the relationships between continuous variables and to predict victimization and aggression, Spearman’s rank correlation and stepwise regression analyses were conducted. This method was selected to identify the most significant predictors in an exploratory approach, given the limited prior literature regarding specific BASC dimensions as predictors in the ASD-L1 population. Regression assumptions were verified. Multicollinearity was ruled out as all Variance Inflation Factors (VIF) were below 2.5. Furthermore, visual inspection of normal P-P plots and scatterplots of standardized residuals versus predicted values confirmed that the assumptions of normality and homoscedasticity of residuals were met. All statistical analyses were performed with a significance level set at *p* < 0.05, and confidence intervals were calculated at the 95% level. Effect sizes were reported using Cohen’s *d* [53] for parametric tests, interpreted as small (*d* ≈ 0.20), medium (*d* ≈ 0.50), and large (*d* ≈ 0.80), and the rank biserial correlation (*r*) for non-parametric tests, interpreted as small (*r* ≈ 0.10), medium (*r* ≈ 0.30), and large (*r* ≈ 0.50).

## 3. Results

### 3.1. Adaptive and Maladaptive Behaviors in ASD-L1

Group comparisons for the three composite dimensions of the BASC S2/S3 (see Table 2) revealed a significant difference for all variables between children and adolescents with ASD-L1 and their TD) peers.

Children and adolescents diagnosed with ASD-L1 reported significantly higher scores of Clinical Maladjustment and School Maladjustment and lower Personal Adjustment than their TD peers, with a small or moderate effect size [53].

### 3.2. Self-Reported Bullying and Victimization

**Figure 1:**
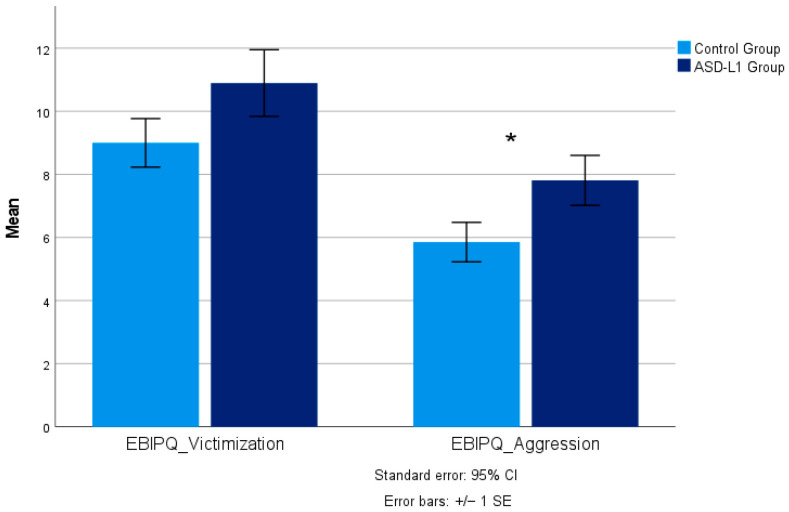
Comparative analysis of the mean values of victimization and aggression between participants with ASD-L1 and the control group (TD peers). Note: Error bars represent Standard Error. * *p* < 0.05 for aggression differences between ASD-L1 and control groups.

Figure 1 presents the results obtained in the EBIP-Q addressing both self-reported victimization and aggression scores.

Results showed no significant differences in self-reported victimization between participants with ASD-L1 (*M* = 10.90, *SD* = 5.48) and their TD peers (*M* = 9.00, *SD* = 5.32), *t*(94) = −1.45, *p* = 0.075, *d* = 0.29. However, children and adolescents with ASD-L1 reported significantly higher levels of aggression (*M* = 7.81, *SD* = 5.48) compared to the control group (*M* = 5.85, *SD* = 4.34), *U* = 844, *p* = 0.022, *r* = 0.23, with a small effect size.

### 3.3. Adaptive and Maladaptive Behaviors as Risk or Protective Factors in Bullying Victimization and Aggression

Correlational analyses (see Table 3) were conducted using Spearman’s rank correlation (Rho, ρ) due to the non-normal distribution of almost all variables. To control for Type I error deriving from multiple comparisons, a Bonferroni correction was applied, establishing a strict significance threshold of *p* < 0.005.

In the ASD-L1 group, victimization and aggression were strongly associated (ρ = 0.642, *p* < 0.001). Regarding adjustment and maladjustment variables, although moderate correlations were observed between higher victimization and lower Personal Adjustment (ρ = −0.354, *p* = 0.014) or higher Clinical Maladjustment (ρ = 0.326, *p* = 0.024), these associations did not reach the adjusted significance threshold after correction. Similarly, no significant associations were found for aggression in this group regarding adaptive or maladaptive behaviors.

In contrast, the TD group showed robust significant associations across almost all dimensions. Both victimization and aggression were significantly correlated with higher Clinical and School Maladjustment and lower Personal Adjustment (all ρ_s_ > 0.41, *p*_s_ ≤ 0.003), suggesting a more generalized link between socio-emotional functioning and bullying involvement in neurotypical adolescents compared to the specific patterns observed in the ASD-L1 group.

Subsequent to this, stepwise regression analyses were performed to ascertain which domains most effectively explained self-reported victimization and aggression in each group. The final predictive models are displayed in Table 4 and Table 5.

As shown in Table 4, different predictors of bullying victimization were identified for each group. In the control group, Clinical Maladjustment accounted for approximately 24% of the variance in self-reported victimization, whereas in the ASD-L1 group, higher Personal Adjustment explained about 17% of the variance in victimization. Thus, distinct underlying mechanisms appear to explain self-perceived victimization among TD students and those with ASD-L1.

Regarding bullying aggression (Table 5), higher School Maladjustment uniquely predicted aggression in the control group, accounting for nearly 23% of its variance. In contrast, a different pattern emerged for the ASD-L1 group, where Clinical Maladjustment was the only significant predictor, explaining 10.5% of the variance. These results indicate that while school-related difficulties are more relevant to aggressive behavior among TD students, internalizing or clinical problems play a greater role in aggression within the ASD-L1 group.

## 4. Discussion

This study sets out to compare adaptive and maladaptive behaviors, as well as bullying victimization and aggression, between children and adolescents with ASD-L1 and their TD peers. It also examined how these behavioral dimensions predicted bullying involvement across groups.

### 4.1. Clinical and Emotional Challenges in Children with ASD-L1

In line with our first hypothesis (H1), children and adolescents with ASD-L1 displayed significantly higher levels of Clinical Maladjustment and School Maladjustment than their TD peers, and lower levels of Personal Adjustment. These results show a general tendency toward poorer adaptive and emotional functioning in the ASD-L1 group. Therefore, these results provide support for H1.

These findings are consistent with extensive evidence indicating that anxiety and related emotional symptoms are highly prevalent among individuals on the autism spectrum, including those with average or above-average cognitive functioning [54,55,56]. Anxiety in autistic youth often manifests in both typical and atypical forms, including heightened distress in response to change, idiosyncratic fears, and excessive worry about social misunderstanding [54,56]. Moreover, anxiety symptoms are not only frequent but also clinically impairing, exerting measurable effects on adaptive functioning [55]. These atypical anxiety presentations, coupled with restricted interests and repetitive behaviors, may contribute to the elevated levels of clinical maladjustment observed in the ASD-L1 group.

In addition, research has shown that adolescents with autism frequently experience social anxiety and diminished self-efficacy in interpersonal contexts [57,58]. Early social communication difficulties may evolve into persistent social anxiety during adolescence, increasing avoidance of peer interactions [57]. Anxiety in youth with autism often arises from difficulties in understanding social norms and predicting others’ behaviors, fostering chronic uncertainty and emotional strain [58]. Such processes may explain the observed lower Personal Adjustment observed in the ASD-L1 group, reflecting a potentially reduced self-esteem and social confidence in this group.

Beyond anxiety, other components of the Clinical Maladjustment composite—namely atypicality, locus of control and somatization—may also contribute to the emotional and behavioral difficulties observed in adolescents with ASD-L1. Previous research has shown that higher levels of Atypicality, associated with unconventional social behaviors and idiosyncratic cognitive styles, are common in individuals with ASD and tend to predict poorer adaptive functioning, social rejection, and lower peer acceptance [59,60]. These findings align with our results, suggesting that social incongruence and difficulties in interpreting contextual norms may underline the elevated clinical maladjustment observed in the ASD-L1 group.

Moreover, differences in perceived control could play a complementary role. Empirical studies indicate that individuals on the autism spectrum often display a more external locus of control, perceiving social and emotional outcomes as governed by external factors rather than personal agency [61,62]. This external orientation has been linked to lower self-efficacy, reduced adaptive coping, and higher stress reactivity [14,61]. Consistent with this interpretation, research on caregivers of autistic children has also found a predominance of external control beliefs, which were associated with poorer psychological adjustment and reduced perceived quality of life [63].

Therefore, the pattern of higher clinical and school maladjustment and lower personal adjustment observed in the ASD-L1 group can be interpreted as a multidimensional construct encompassing atypical social behavior, externalized control beliefs, and emotional dysregulation, features that jointly shape the socio-emotional vulnerability characteristic of this population.

These socio-emotional difficulties may, in turn, heighten susceptibility to bullying and social exclusion, as emotional dysregulation and misinterpretation of social cues can elicit maladaptive responses in peer interactions.

### 4.2. Bullying and Aggression

Regarding our second hypothesis (H2), which predicted higher levels of both bullying victimization and aggression among the ASD-L1 group, the findings provided only partial support. Participants with ASD-L1 reported higher mean levels of both forms of bullying involvement compared to their TD peers; however, this difference reached statistical significance only for aggression. This pattern suggests that, whilst adolescents with ASD-L1 have been shown to perceive greater overall engagement in bullying dynamics, this was primarily driven by elevated self-reported aggressive behaviors, rather than increased victimization experiences.

As indicated by the extant literature, there is a consistent emphasis on the elevated vulnerability of autistic youth regarding peer conflict and involvement in bullying. However, it should be noted that the results of such studies have been found to vary depending on methodological and contextual factors. Large-scale studies have reported increased rates of both victimization and aggression among students on the autism spectrum compared with their peers [15,64]. However, other investigations have indicated that the extent of victimization may be contingent on the school environment, peer awareness, and teacher attitudes towards inclusion [16,65]. In this sense, the absence of significant between-group differences in victimization in the present study may reflect protective contextual factors, such as inclusive educational practices or improved social understanding of autism among peers.

Beyond the school context, broader cultural and systemic influences may also shape how bullying is experienced and reported. International frameworks such as the Programme for International Student Assessment (PISA 2022) have underscored that bullying is closely linked to students’ socio-emotional well-being and overall school climate across countries [66,67]. From this systemic perspective, the patterns observed in our study may be better understood within wider educational and cultural contexts, where social support and inclusive practices could mitigate the effects of vulnerability.

About aggression, the markedly elevated levels of self-reported aggression observed in the ASD-L1 group are consistent with prior research indicating that aggressive behaviors in individuals with autism are predominantly reactive rather than proactive [28,68]. Such behaviors are typically associated with emotional dysregulation, frustration, and difficulties interpreting social cues, rather than with deliberate hostility. Rieffe [21] demonstrated that emotion dysregulation mediates the relationship between social stress and aggression in adolescents with ASD. Matson and Adams [68] also observed that aggression in students with autism frequently arises as a reaction to misunderstanding or perceived provocation, rather than as an intentional act of dominance. These findings are consistent with the present results, suggesting that increased aggression among adolescents with ASD-L1 may stem from emotional and regulatory difficulties rather than from proactive antisocial intent.

Taken together, these findings indicate that while the ASD-L1 group does not necessarily experience higher victimization rates, their emotional and behavioral profile may predispose them to greater reactive aggression. This pattern underscores the necessity for diversified intervention strategies, emphasizing not solely the prevention of peer victimization but also the enhancement of emotion regulation, social comprehension, and coping mechanisms to mitigate maladaptive aggressive responses. The enhancement of these socio-emotional competencies has the potential to curtail conflict escalation and to nurture more positive peer relationships in adolescents diagnosed with ASD-L1.

### 4.3. Risk and Protective Factors of Bullying Experiences

The findings partially supported our third hypothesis (H3), in which we proposed that adaptive and maladaptive indicators would be more strongly associated with frequent bullying experiences in the ASD-L1 group, and that they would act as either protective or risk factors in explaining bullying involvement, both in terms of victimization and aggression. Accordingly, correlational analyses were first conducted to examine associations between the adjustment composites and both forms of bullying involvement, which were subsequently analyzed through regression models to identify the most relevant predictors within each group.

#### 4.3.1. Correlational Analyses

We found associations between adjustment indices and bullying in both groups, though with different statistical robustness. In the TD group, correlations were widespread and highly significant (see Table 3), suggesting a generalized link between socio-emotional functioning and peer dynamics. In contrast, within the ASD-L1 group, although lower Personal Adjustment was related to greater victimization and Clinical Maladjustment to aggression with moderate effect sizes, these associations did not survive the strict Bonferroni correction (*p* < 0.005). This suggests that while the link exists, it may be less linear or more heterogeneous in adolescents with ASD-L1 compared to their TD peers. However, despite the loss of significance in bivariate correlations due to conservative corrections, the regression analyses (which account for shared variance) successfully identified specific predictors, as discussed below.

#### 4.3.2. Predictors of Bullying Experiences

Regression analyses showed that adaptive and maladaptive indicators acted as protective or risk factors in explaining bullying involvement, but the results revealed distinct predictive patterns underlying aggression and victimization between groups.

*Predictors of bullying experiences in TD youth:* For victimization scores, higher clinical maladjustment (characterized by increased anxiety, atypicality, and externalized control beliefs) emerged as a risk factor of vulnerability to bullying. This pattern suggests that emotional instability, social incongruence, and a diminished sense of control may heighten adolescents’ perceived helplessness in peer interactions, thereby increasing their susceptibility to victimization. It is also plausible that higher levels of internalizing problems stem from more extensive episodes of victimization experienced. Similarly, in TD youth, bullying aggression appears to be primarily shaped by contextual and behavioral factors, as higher school maladjustment predicted aggression, suggesting that external stressors within the school environment and tendencies toward externalizing behavior play a central role in these dynamics. This aligns with evidence showing that academic difficulties, poor teacher–student relationships, and negative school climate can exacerbate aggressive responses in adolescents without neurodevelopmental conditions [69,70].

*Predictors of bullying experiences in ASD-L1 youth:* In terms of bullying victimization, personal adjustment operated as a protective factor, indicating that stronger self-esteem, positive family and peer relationships, and greater self-reliance can compensate for the negative effects of social stress and reduce exposure to bullying victimization. In contrast to the TD peers, in the ASD-L1 group, aggression was more closely associated with clinical maladjustment, reflecting internal distress and emotional dysregulation. This pattern indicates that aggressive behaviors may arise from frustration, anxiety, or difficulties in emotion regulation rather than from deliberate hostility. Similar findings have been reported in prior studies linking emotional instability and internalizing symptoms to reactive aggression in autistic youth [14,71].

The current findings of this study emphasize the qualitative differences in risk and protective mechanisms between the groups. For TD participants, emotional insecurity and perceived lack of control appear to intensify vulnerability to peer aggression, whereas for individuals with ASD-L1, a better adaptive functioning (particularly in domains related to interpersonal and self-perceptive adjustment) mitigates this risk. These results underscore the importance of fostering supportive relationships, resilience, and a positive self-concept particularly (but not solely) in students with ASD-L1 to enhance their socio-emotional competence and prevent victimization.

These results align with and extend previous research emphasizing the relevance of internalizing difficulties and adaptive functioning in explaining bullying involvement among individuals with ASD-L1. Consistently, studies by Zablotsky [72] and Sterzing [64] identified behavioral problems as significant risk factors for bullying in children with ASD. In this regard, several studies have further examined how emotional dysregulation, social vulnerability, and self-perception interact to shape bullying dynamics in this population. In this vein, Little [73] emphasized that social vulnerability is a significant predictor of bullying among children with ASD, surpassing factors such as behavioral problems and anxiety. These findings suggest that social vulnerability—characterized by difficulties in social interaction and being perceived as different—may be a pivotal factor contributing to bullying in ASD-L1 populations. Incorporating this construct as a key variable in future research could advance a more comprehensive understanding of the risk factors associated with bullying in this group.

Conversely, enhanced personal adjustment appears to serve as a protective factor against bullying victimization for ASD-L1 participants. Although a similar negative correlation was observed in TD peers, personal adjustment did not emerge as a predictor in suggesting that this protective mechanism may operate more prominently among individuals with ASD-L1. This finding highlights the crucial role of adaptive functioning in mitigating the emotional impact of social interactions and reducing susceptibility to bullying, aligning with prior evidence linking greater social competence and self-perceived adjustment to lower levels of peer victimization in autistic populations [16,25,30].

Importantly, this pattern reflects the broader construct of personal adjustment rather than self-confidence alone. While self-confidence is often cited as a protective factor in the general population, its role may be limited in ASD-L1 individuals due to challenges in self-awareness, introspection, and emotional regulation [61]. These results highlight the need for individualized interventions that strengthen not only self-esteem but also the underlying cognitive and emotional mechanisms that support adaptive functioning and resilience in ASD-L1 profiles [16,61,62].

The finding suggests a potential link between maladaptive behaviors (clinical maladjustment) and increased vulnerability to bullying experiences [64]. However, given the correlational and cross-sectional nature of the study, it is not possible to draw causal inferences. It is also plausible that bullying experiences might exacerbate existing emotional and behavioral challenges, suggesting a potentially bidirectional relationship that should be explored in future longitudinal research [28,64]. In this vein, because clinical maladjustment predicted aggression in the ASD-L1 group, their aggressive behaviors are not closely linked to academic or contextual difficulties, as observed in their neurotypical peers [69,70], but rather stem from internal emotional distress [14,71]. Such patterns indicate that aggression in ASD-L1 may arise more directly from emotional dysregulation and difficulties in processing affective states [14,71,74], rather than from external factors such as academic challenges, school maladjustment, or social self-perception.

In a different pattern, school maladjustment was identified as a predictor of aggression in TD peers, reflecting a more externalizing behavioral pattern [69,70]. These findings emphasize the distinct socio-emotional pathways underlying bullying and aggression in ASD-L1 compared with TD peers. In sum, while aggression in neurotypical adolescents appears to be primarily driven by contextual and behavioral stressors, aggression in ASD-L1 youth seems to emerge from internal emotional dysregulation and difficulties in processing affective states [14,71]. In this population, aggressive behaviors might thus be less connected to self-esteem, self-concept, interpersonal relations, or even academic difficulties and might instead stem from difficulties in emotional regulation and negative affectivity.

The Social-Information Processing (SIP) model, as proposed by Crick and Dodge [74], offers a useful explanatory framework for interpreting these results. According to this model, individuals who experience difficulties in encoding, interpreting, or responding to social cues are more likely to misperceive ambiguous interactions as hostile, which can lead to reactive and maladaptive behavioral responses such as aggression. This theoretical perspective has received consistent empirical support in autism research, with studies by Zablotsky and Sterzing [64,72] demonstrating that atypical social cue processing and limited emotion understanding contribute to increased social conflict and aggressive behaviors in individuals with ASD. These observations are also consistent with previous research [75,76], highlighting the influence of emotional difficulties, poor regulation, and comorbid conditions (such as anxiety or attention problems) on the manifestation of both victimization and aggression in children with ASD.

### 4.4. Novel Contributions and Implications

A distinctive feature of the present study is its focus on individuals diagnosed with ASD-L1. This group is frequently overlooked in research when compared to studies on ASD in general [15,25,77]. Recent studies have begun to address ASD-L1 specifically, thus reinforcing the need to investigate this subgroup independently [78,79,80,81]. By focusing on ASD-L1, our research provides novel insights into the adaptive and maladaptive behaviors and bullying experiences of this population, contributing to the emerging literature that differentiates ASD-L1 from broader ASD samples.

However, while this study is among the few to center explicitly on individuals diagnosed with ASD-L1 according to prevailing diagnostic criteria, it is essential to recognize that prior research on Asperger’s Syndrome or HFA may have comprised participants exhibiting comparable profiles. Consequently, although the present findings offer a novel contribution framed within the ASD-L1 classification, overlapping with earlier samples must be considered when interpreting comparisons with past studies.

Another notable aspect of our study is the examination of the diverse etiologies and pathways and origins of aggressive behaviors in individuals with ASD-L1 relative to their TD counterparts. These findings suggest that aggression observed in individuals with ASD-L1 may be primarily associated with emotional dysregulation, whereas in the control group it appears to be linked to school-related maladjustment. This insight offers a novel contribution to literature by highlighting how the underlying mechanisms of aggression may differ between these groups. Furthermore, the focus on a Spanish-speaking population represents a valuable addition to the research, addressing a group that has been underrepresented in the existing literature.

Our research has several educational implications, since it highlights the necessity for the development of educational programs that foster an awareness of inclusion and diversity. Training school personnel and students in empathy and bullying management skills can positively impact the school environment and reduce instances of victimization. Moreover, it is of paramount importance to provide tailored assistance to individuals with ASD-L1, with the aim of facilitating the management of their emotions and the reduction in aggressive behaviors. It is recommended that interventions focus on enhancing social and emotional skills, emotion regulation, and stress management strategies, which are vital in mitigating aggression and increasing emotional competence. To comprehensively address the needs of individuals with ASD-L1, it is essential to promote interdisciplinary collaboration among psychologists, therapists, and educators. This will ensure that individuals receive the necessary tools to handle emotional challenges effectively and foster a positive social environment.

### 4.5. Limitations and Future Directions

One limitation of the present study is the exclusive reliance on self-report measures, which may have introduced common method variance and potentially inflated some of the observed associations. However, to address this concern, we conducted Harman’s single-factor test. The results revealed that the first unrotated factor accounted for only 16.57% of the total variance, falling well below the critical 50% threshold. This finding suggests that common method bias is not a significant concern in the present study and supports the discriminant validity of the constructs assessed.

Nevertheless, the utilization of validated self-report instruments in individuals with ASD-L1 can also be regarded as a strength, as these tools capture the subjective perspective of those directly affected, which may not always coincide with parental or teacher reports [36,39]. It is recommended that future research incorporate multi-informant designs (e.g., parents, teachers, peers) and employ more fine-grained subscales to disentangle the specific contribution of each dimension to bullying involvement in ASD-L1.

Additionally, it would be beneficial for future studies to consider larger samples and more diverse and complementary assessment methods and variables, integrating diverse approaches such as direct observation and third-party reports, to validate these findings, as well as the exploration of social vulnerability as a risk factor for bullying experiences [80,81,82]. In terms of methodology, future research would move beyond cross-sectional approaches and advance toward longitudinal designs. The implementation of longitudinal designs would facilitate the determination of the directionality of the relationships between maladjustment and bullying over time. Moreover, to address the inherent limitations of observational comparisons between clinical and control groups, the use of quasi-experimental techniques, such as propensity score matching, would be recommended to better control for potential confounding variables.

It would also be beneficial to explore specific interventions designed to reduce socio-emotional difficulties and decrease the incidence of bullying in this group. Future research might also focus on investigating the effectiveness of interventions aimed at improving emotion regulation and social skills in individuals with ASD-L1.

Finally, the absence of systematic documentation pertaining to support services (e.g., speech therapy, educational accommodation) constitutes an additional limitation. While this omission does not fundamentally challenge the primary findings, it does impose limitations on the contextual interpretation of the data. This issue must be addressed in future research designs to ensure the robustness of the results obtained.

## 5. Conclusions

This study highlights the complexity of socio-emotional functioning and bullying involvement in children and adolescents with ASD-L1. Children with ASD-L1 exhibited greater clinical and school maladjustment and lower personal adjustment compared to their TD peers. Although no significant differences were found in bullying victimization, they showed higher levels of aggression, suggesting a distinct behavioral profile that may contribute to their involvement in bullying dynamics. Correlational analyses revealed robust association patterns between socio-emotional adjustment and bullying involvement in the TD group, whereas these associations were less consistent in the ASD-L1 group after correcting for multiple comparisons. This suggests that the mechanisms underlying peer dynamics may be more heterogeneous in autistic youth. Furthermore, the specific predictors identified in the regression models indicate that the sources of these associations differ in nature.

Taken together, the present findings indicate that personal adjustment, encompassing self-esteem, confidence, and interpersonal relations, acts as a protective factor against bullying victimization particularly in children and adolescents with ASD-L1. Meanwhile, aggression in this group is primarily related to clinical maladjustment, reflecting an internal clinical base characterized by anxiety, atypicality, locus of control, and somatization. In contrast, clinical maladjustment acts as a predictor of bullying victimization, not aggression in neurotypical participants while aggression in this group appears to be more linked to negative attitudes towards school and teachers and a tendency towards sensation seeking.

These results underscore the necessity for intervention programs to promote clinical stabilization, school engagement, and the development of adaptive personal competencies in the school setting.

## Figures and Tables

**Table 1:** Descriptive statistics by group of the initial screening tests (N = 96).

	n	ASD-L1 Mean (*SD*)	Control Mean (*SD*)	*t* (*p*)	*d*
Age	96	9.98 (1.88)	10.10 (1.85)	0.327 (0.074)	0.06
SES	96	3.31 (0.854)	3.23 (0.857)	−0.477 (0.63)	0.09
Raven (total score)	96	110.90 (12.33)	111.50 (13.02)	0.233 (0.81)	0.04
ASSQ	88	34.48 (9.70)	7.86 (5.02)	**16.02 (<0.001)**	−3.41
ASSQ-Girl	8	7.50 (4.12)	4.5 (3.96)	2.17 (0.07)	0.75
AQ-Child & Adolescent	96	63.58 (11.02)	31.75 (14.73)	**11.98 (<0.001)**	−2.44

Note. SES: Socioeconomic Status [1: lowest level; 6: highest level] [48]; ASSQ: Autism Spectrum Screening Questionnaire [43]; AQ: Autism Spectrum Quotient [44,45]; *SD*: Standard Deviation; *p*: significance level; *d*: effect size with Cohen’s d; Bold depicts significant group differences.

**Table 2:** Adaptive and maladaptive behaviors between children and adolescents with ASD-L1 and TD according to BASC S2/S3 dimensions.

		ASD-L1		Control		*U*	*p*	*r*
	*n*	*M*	*SD*	*M*	*SD*			
Clinical maladjustment	96	19.73	9.66	14.59	10.71	801.5	0.010 *	0.26
School maladjustment	96	4.89	3.61	3.72	4.24	832.5	0.019 *	0.24
Personal adjustment	96	29.0	4.47	31.36	7.45	643.5	<0.001 **	0.38

Note: * *p* < 0.05; ** *p* < 0.01; BASC (Behaviour Assessment System for Children) [11].

**Table 3:** Correlation matrix between BASC Scales and EBIPQ Victimization and Aggression scores for the control (lower diagonal) and the ASD-L1 group (upper diagonal).

	ASD-L1 Group	1	2	3	4	5
Control Group						
(1) EBIPQ Victimization		1	0.642 **	0.326	0.292	−0.354
(2) EBIPQ Aggression		0.717 **	1	0.188	0.245	−0.147
(3) Clinical maladjustment		0.503 **	0.419 *	1	0.411 *	−0.342
(4) School maladjustment		0.478 **	0.520 **	0.644 **	1	−0.392
(5) Personal adjustment		−0.547 **	−0.420 *	−0.518 **	−0.529 **	1

Note: * *p* < 0.05; ** *p* < 0.01 (Bonferroni corrected).

**Table 4:** Stepwise regressions predicting victimization from the BASC scales for the Control and ASD-L1 groups, respectively.

Dependent: EBIPQ-Victimization				
	Control group			
Predictor	β	Adj.*R*^2^	*F* or *t*	*p*
Step 1		0.237	15.566	0.000
Clinical Maladjustment	0.503		3.945	0.000
	ASD-L1 Group			
Predictor	β	Adj.*R*^2^	*F* or *t*	*p*
Step 1		0.169	10.584	0.002
Personal Adjustment	−0.432		−3.253	0.002

Note: β: Standardized regression; Adj. R^2^: Adjusted R-squared; *F* or *t*: represents the *F*-statistic from an ANOVA; *t* refers to *t*-statistic; *p* < 0.05. No collinearity issues were detected (all VIF < 2.5).

**Table 5:** Regression Analysis Predicting EBIPQ aggression from BASC scales for the Control and ASD-L1 groups, respectively.

Dependent: EBIPQ Aggression				
	Control group			
Predictor	β	Adj.R^2^	*F* or *t*	*p*
Step 1		0.226	14.690	0.000
School maladjustment	0.492		3.833	0.000
	ASD-L1 Group			
Predictor	β	Adj.R^2^	*F* or *t*	*p*
Step 1		0.105	6.493	0.014
Clinical Maladjustment	0.352		2.548	0.014

Note: β: Standardized regression coefficient; Adj. R^2^: Adjusted R-squared; *F* or *t*: represents the F-statistic from an ANOVA; *t* refers to *t*-statistic; *p* < 0.05; No evidence of collinearity was found (all VIF < 2.5).

## Data Availability

The data supporting the findings of this study are available on request from the corresponding author. The data are not publicly available because it contains information that could compromise the privacy of research participants.

## Abbreviations

The following abbreviations are used in this manuscript:

ASD	Autism Spectrum Disorder
ASD-L1	Autism Spectrum Disorder Level 1
ASSQ	Autism Spectrum Screening Questionnaire
AQ	Autism Spectrum Quotient (Child/Adolescent versions)
BASC	Behavior Assessment System for Children
EBIP-Q	European Bullying Intervention Project Questionnaire
SES	Socioeconomic Status
ADOS-2	Autism Diagnostic Observation Schedule, Second Edition
ADI-R	Autism Diagnostic Interview, Revised
SD	Standard Deviation
SE	Standard Error
β	Standardized regression coefficient
Adj. R^2^	Adjusted R-squared
PISA	Programme for International Student Assessment
OECD	Organisation for Economic Co-operation and Development
UIB	Universitat de les Illes Balears/University of the Balearic Islands
UNAL	Universidad Nacional de Colombia/National University of Colombia
